# Descriptive Epidemiology of COVID-19 in Hokkaido, Japan: Regional Burden and the Role of Railway-Driven Population Mobility

**DOI:** 10.7759/cureus.84208

**Published:** 2025-05-16

**Authors:** Yuji Kaneko

**Affiliations:** 1 General Medicine, HITO Medical Center, Ehime, JPN

**Keywords:** age-standardized incidence rates, covid-19, descriptive epidemiology, population mobility, railway transportation

## Abstract

Background

Understanding regional disparities in COVID-19 incidence and the factors influencing them is essential for effective public health responses. In Japan, particularly in Hokkaido, significant differences in case burden have been observed across municipalities. This study aimed to describe the epidemiological patterns of COVID-19 in Hokkaido by adjusting for age structure and to examine the association between railway-based population mobility and regional case burden.

Method

We categorized all municipalities in Hokkaido into five public health center jurisdictions: Sapporo, Otaru, Asahikawa, Hakodate, and Other Areas. COVID-19 incidence was assessed across the third to sixth waves of the pandemic (November 2020 to June 2022). To account for demographic differences, age-standardized incidence ratios (SIR) were calculated using national age-specific incidence rates and the indirect standardization method. Data on confirmed cases were obtained from government open datasets, and population mobility was measured using railway transport density from the Hokkaido Railway Company. Statistical analyses included chi-square tests and multiple regression analysis, including both full and simplified models to assess statistical associations, with the latter addressing multicollinearity among demographic variables.

Results

A total of 378,281 cases were reported during the study period, with the highest burden in Sapporo. SIR exceeded 1.0 in Sapporo, Otaru, and Asahikawa during multiple waves, indicating higher-than-expected incidence after age adjustment. Notably, Sapporo exhibited elevated SIR, particularly among older adults, while Otaru and Asahikawa showed higher rates among those aged 20-50 years. In regression analyses, railway transport density showed a statistically significant positive association with SIR in both univariable (p = 0.002) and simplified multivariable models that included the effective reproduction number (Rt). In the multivariable model, railway transport density remained positively associated (p < 0.001), while Rt showed a negative association (p = 0.032), with an adjusted R² of 0.524. These findings suggest that railway-based mobility and real-time transmission dynamics jointly influence regional disparities in COVID-19 burden.

Conclusion

This study highlights the significant impact of railway-based population mobility on COVID-19 transmission within Hokkaido, Japan. Age-standardized comparisons revealed regional variations in risk, likely influenced by urban proximity and transport infrastructure. These findings support the incorporation of geographic and mobility-related metrics into geographically tailored public health policies. Further research using larger datasets and multimodal transport indicators is recommended to validate and expand upon these insights.

## Introduction

The COVID-19 pandemic, which began in January 2020, has underscored the critical importance of learning from past outbreaks to prepare for future pandemics [1-3]. Retrospective analyses of regional outbreaks can offer valuable insights into effective intervention strategies for infectious disease control [4].

In Japan, as in many countries, the severity of the COVID-19 pandemic varied significantly across regions due to factors such as population density, age distribution, and patterns of human mobility [5,6]. Public transportation, particularly railways, plays a major role in daily life and commuting in Japan, especially in urban areas, and has been recognized as a key factor influencing the geographic spread of the virus [7-9].

Hokkaido, the northernmost island of Japan and the country’s largest prefecture by area, presents a unique case for epidemiological analysis due to its diverse geography and population distribution. Throughout the pandemic, Sapporo - the largest city in Hokkaido - consistently reported the highest number of COVID-19 cases. Other urban centers, such as Asahikawa and Hakodate, also experienced significant case numbers [10]. However, direct comparison of case counts across regions without adjusting for differences in population size and age structure may lead to misleading conclusions.

While several studies in Japan have examined the pandemic at the prefectural level, there remains a lack of detailed analysis at the municipal level that accounts for demographic factors. Furthermore, the role of intra-prefectural mobility, particularly via rail transport, has not been thoroughly evaluated in this context.

This study aims to address these gaps by recalculating the incidence rates of COVID-19 across municipalities in Hokkaido, adjusting for age structure, and examining how changes in railway-based population mobility influenced regional infection trends. The findings are intended to contribute to more nuanced infectious disease policy development, both within Japan and in other regions with similar transportation and demographic characteristics.

## Materials and methods

First, all municipalities within Hokkaido were categorized into five groups based on their public health center jurisdictions: “Sapporo,” “Otaru,” “Asahikawa,” “Hakodate,” and “Other Areas” (Table 1).

**Table 1:** Classification of municipalities, included areas, and total population in Hokkaido Source: Statistics division, policy planning department, Hokkaido government [11]

Region	Population	Population density (persons/km²)
Sapporo	1,973,395	1760.0
Otaru	111,299	456.5
Asahikawa	329,306	440.4
Hakodate	251,084	370.4
Other Areas	2,559,530	31.7

The incidence rate (defined as the number of confirmed positive cases divided by the population) was calculated for each region. The study period was divided into four distinct waves of the pandemic (Table 2). 

**Table 2:** COVID-19 pandemic waves and their time periods The definition of the pandemic period is based on calculations made in accordance with the verification report “Directions for Responding to New Infectious Disease Crises in Hokkaido” published by the Hokkaido Government in December 2023 [12].

Pandemic wave	Period
3rd wave	November 1, 2020 – February 28, 2021
4th wave	March 1, 2021 – June 30, 2021
5th wave	July 1, 2021 – December 31, 2021
6th wave	January 1, 2022 – June 30, 2022

To adjust for differences in age distribution across regions, age standardization was performed using the national age-specific incidence rates derived from the national COVID-19 case database, HER-SYS (Health Center Real-time Information-sharing System on COVID-19) [13]. Using these data, the standardized incidence ratios (SIR) was calculated for each region.

The SIR was computed using the indirect standardization method. The expected number of infected cases (I_E_*)* for each population was calculated based on the age-specific incidence observed nationwide. I_E_ and SIR were calculated using equations (1) and (2). Age groups were categorized in 10-year intervals. It was assumed that COVID-19 vaccination coverage was equivalent across all regions in Hokkaido and the national average.

$I_E = \sum n_a N_a i / N_a$ (1)

$SIR = \frac{I_O}{I_E}$ (2)

*na *: Population of the age group in the study region

*Nai *: Number of newly confirmed cases in the age group nationwide

*Na *: National population of the age group

*Io *: Observed number of infected cases

Data sources and statistical analysis

For this study, data on newly confirmed COVID-19 cases were obtained from press release materials and open datasets published by the Hokkaido Prefectural Government [14]. Population data were based on the 2020 Population Census of Japan [11]. The susceptible population was defined as the total population of each region. For cases where age data were not disclosed, an age distribution was estimated based on national age-specific incidence rates.

To evaluate regional differences in the SIR of COVID-19, chi-square tests were conducted to compare the observed number of cases in each region with the expected number, which was calculated based on regional population proportions. The test assessed whether the distribution of cases significantly deviated from expectations under the assumption of uniform risk across regions. Bonferroni correction was applied to adjust for multiple comparisons, with a significance threshold set at p < 0.005. These tests were performed separately for each pandemic wave to assess temporal variation in regional disparities.

Subsequently, the impact of changes in population mobility before and during the pandemic on SIR was examined using multiple regression analysis. As an indicator of intercity population mobility, we used “Railway transport density” (measured in persons per kilometer per day), a metric published by the Hokkaido Railway Company [15]. Other explanatory variables included the effective reproduction number (Rt), population density, average household size, proportion of elderly population, and proportion of working-age population. Rt is a commonly used metric indicating the average number of secondary infections caused by a single case. The model was fitted using ordinary least squares (OLS), and multicollinearity was assessed using the variance inflation factor (VIF). To assess the relationship between population mobility and COVID-19 incidence, we prioritized simplified regression models using railway transport density as the primary explanatory variable, given its conceptual relevance and lower collinearity with demographic covariates. A univariable linear regression model was first constructed to evaluate the direct association between railway transport density and the standardized incidence ratio (SIR). In addition, a reduced multivariable model was developed that included both railway transport density and Rt. These models were selected based on their conceptual alignment with disease spread mechanisms and their statistical stability.

**Figure 1:**
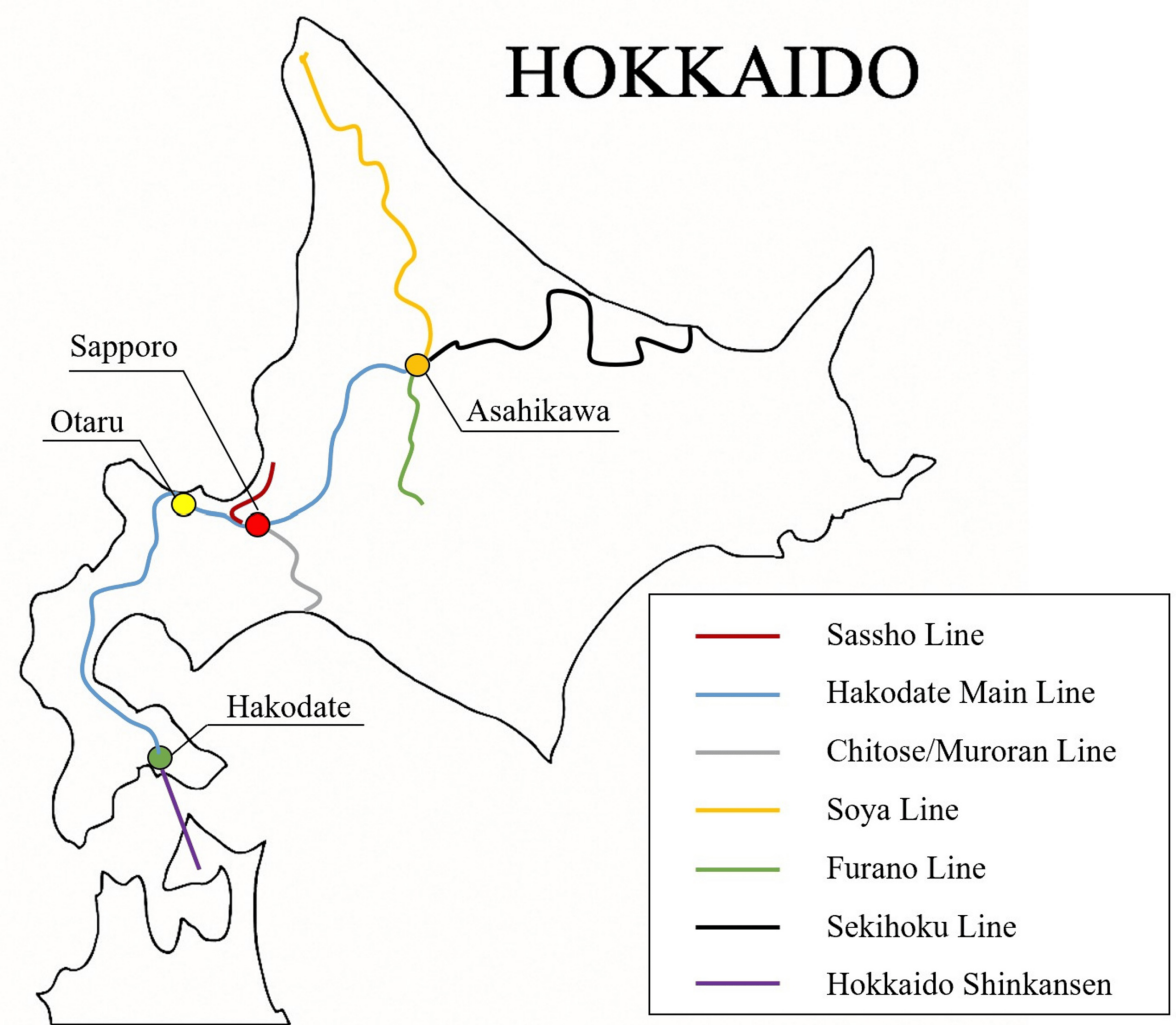
Geographical relationship of major cities and railway lines in Hokkaido This figure was created by the authors based on information from the official website of the Hokkaido Railway Company [16].

For the Sapporo metropolitan area, transport density was calculated as the sum of three lines connected to the city: the Sassho Line, the Hakodate Main Line, and the Chitose/Muroran Line (Figure 1). 

The Otaru area included the Hakodate Main Line. The Asahikawa area included the Soya Line, the Hakodate Main Line, the Furano Line, and the Sekihoku Line. The Hakodate area included the Hokkaido Shinkansen and the Hakodate Main Line.

Changes in transport activity were calculated as a ratio relative to fiscal year 2019 (pre-pandemic baseline), referred to as the "transport density ratio" in this study.

Regarding travel time by rail from Sapporo to other cities, the shortest durations were as follows (based on publicly available timetables from the Hokkaido Railway Company) (Table 3) [15].

**Table 3:** Travel time by rail from Sapporo to other cities (based on Hokkaido Railway Company timetables) Hokkaido Railway Company timetables [15]

Destination	Travel time	Train type
Otaru	25 minutes	Local train
Asahikawa	85 minutes	Limited express
Hakodate	210 minutes	Limited express

Statistical analyses were conducted using the scipy.stats module in Python version 3.9.7. This study utilized only publicly available open data and did not involve any handling of personal or identifiable information.

Ethics statement

This study exclusively utilized publicly available and fully anonymized datasets. As such, it did not involve any direct contact with human subjects, nor did it include any personally identifiable information. Accordingly, institutional review board (IRB) approval and informed consent were not applicable.

## Results

The total number of newly confirmed COVID-19 cases in Hokkaido during the study period was 378,281. Of these, 185,016 cases were reported in Sapporo, 7,930 in Otaru, 23,806 in Asahikawa, and 15,992 in Hakodate. The remaining 145,537 cases occurred in other areas of Hokkaido. I_O_, I_E_, and SIR for each region and each pandemic wave are shown in Table 4. 

**Table 4:** SIR of COVID-19 in different regions of Hokkaido, Japan *Significant at the Bonferroni-adjusted significance level (5%: p = 0.005) SIR: standardized incidence ratios

Period	Region	Observed cases	Expected cases	SIR	p-value*	Degrees of freedom	Cramér's V
3^rd^ wave	Hokkaido	15,958	-	-	-	-	-
	Sapporo	9,301	6,169	1.5	p＜0.001	4	0.2467
	Otaru	566	329	1.7			
	Asahikawa	1,536	984	1.6			
	Hakodate	424	752	0.6			
	Other Areas	4,131	7,724	0.5			
4^th^ wave	Hokkaido	22,209	-	-	-	-	-
	Sapporo	14,463	8,705	1.7	p＜0.001	4	0.2689
	Otaru	394	447	0.9			
	Asahikawa	944	1,362	0.7			
	Hakodate	322	1,036	0.3			
	Other Areas	6,086	10,688	0.6			
5^th^ wave	Hokkaido	20,134	-	-	-	-	-
	Sapporo	11,927	8,192	1.5	p＜0.001	4	0.2198
	Otaru	456	371	1.2			
	Asahikawa	1,780	1,208	1.5			
	Hakodate	586	905	0.6			
	Other Areas	5,385	9,493	0.6			
6^th^ wave	Hokkaido	319,980	-	-	-	-	-
	Sapporo	149,325	127,084	1.2	p＜0.001	4	0.0767
	Otaru	6,514	5,910	1.1			
	Asahikawa	19,546	19,586	1.0			
	Hakodate	14,660	14,357	1.0			
	Other Areas	129,935	153,322	0.8			

Chi-square tests revealed statistically significant differences in SIR between regions during each pandemic wave (p < 0.001 for all waves). During the third wave, SIR exceeded 1.0 in Sapporo (1.5), Otaru (1.7), and Asahikawa (1.6), whereas Hakodate (0.6) and Other Areas (0.5) remained below 1.0. In the fourth wave, only Sapporo had an SIR above 1.0 (1.7) while Otaru (0.9), Asahikawa (0.7), Hakodate (0.3), and Other Areas (0.6) were all below 1.0. During the fifth wave, SIR once again exceeded 1.0 in Sapporo (1.5), Otaru (1.2), and Asahikawa (1.5). Hakodate (0.6) and Other Areas (0.6) remained low. In the sixth wave, Sapporo (1.2) and Otaru (1.1) had slightly elevated SIRs, while Asahikawa and Hakodate were around 1.0, and Other Areas remained below 0.8. Throughout the study period, Hakodate and Other Areas consistently showed SIR values at or below 1.0 in all waves.

**Figure 2:**
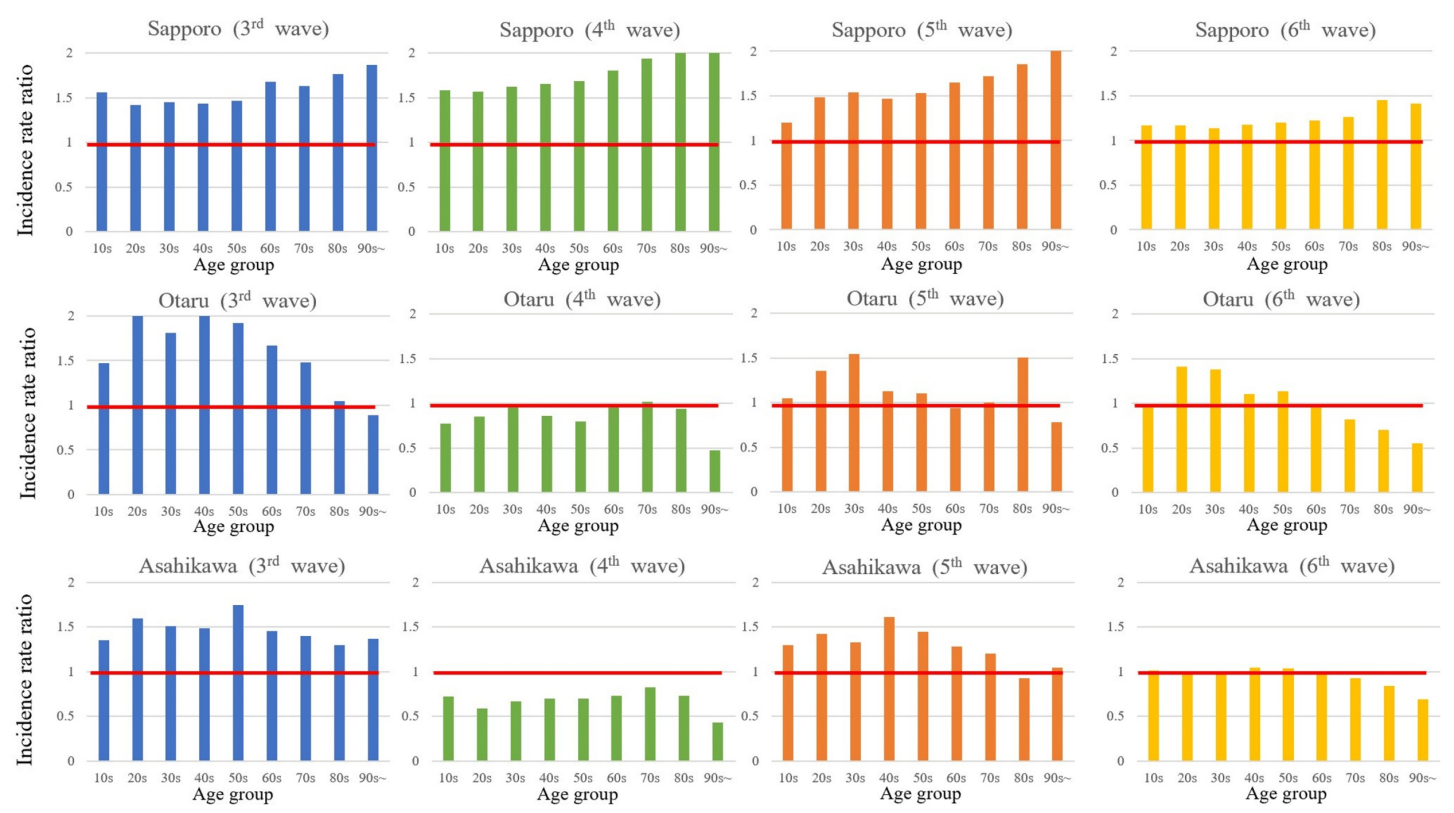
Age-specific incidence rate ratios for each pandemic wave

Focusing on the three cities where SIR exceeded 1.0 - Sapporo, Asahikawa, and Otaru - Figure 2 illustrates the age-specific incidence rate ratios during each pandemic wave.

In Sapporo, higher incidence rate ratios were consistently observed among older age groups across all waves. In contrast, Otaru and Asahikawa showed peak incidence rate ratios among individuals in their 20s to 50s.

**Figure 3:**
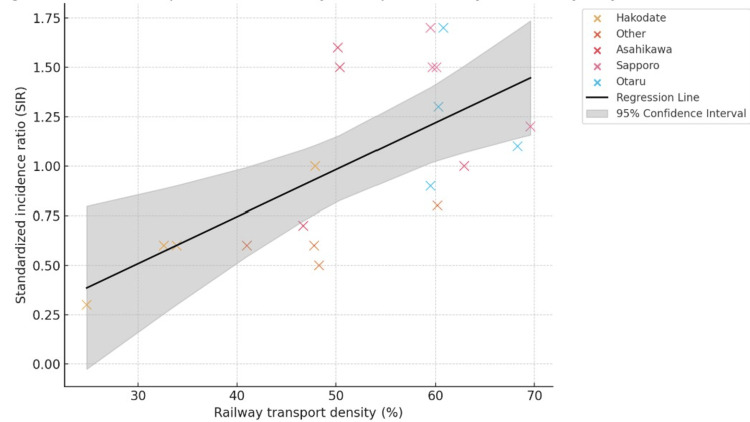
Relationship between railway transport density ratio and SIR by city This figure illustrates the association between railway transport density and the SIR of COVID-19 across 20 observations, categorized by city. The black line indicates the linear regression fit based on a univariable model, while the shaded area represents the 95% confidence interval of the predicted values. A statistically significant positive association was observed (β = 0.0237, p = 0.002; Adjusted R² = 0.405), suggesting that increased rail-based population mobility may contribute to elevated regional COVID-19 burden. SIR: standardized incidence ratios

To address concerns regarding the stability of coefficients in the presence of multicollinearity, we prioritized results from simplified regression models that excluded highly correlated demographic variables. To assess the independent effect of railway-based population mobility on COVID-19 incidence, we conducted a linear regression analysis using railway transport density as a single explanatory variable. This model was chosen to avoid the instability caused by severe multicollinearity among demographic covariates, as indicated by variance inflation factors (VIF) exceeding acceptable thresholds. Rather than arbitrarily excluding certain variables, we adopted a minimalist model to isolate the mobility effect under stable statistical assumptions. The model showed a statistically significant positive association between railway transport density and SIR (β = 0.0237, p = 0.002; Adjusted R² = 0.405), indicating that higher levels of rail-based mobility were linked to increased regional incidence (Figure 3).

A simplified multivariable model incorporating both railway transport density and Rt also demonstrated statistically significant results. Railway transport density remained positively associated with SIR (β = 0.0297, p < 0.001), while Rt was negatively associated (β = -23.2548, p = 0.032). The model's fit improved, with an adjusted R² of 0.524.

## Discussion

In this study, we categorized the regions of Hokkaido based on public health center jurisdictions and calculated the SIR for each area using data from press releases and open datasets provided by the Ministry of Health, Labour and Welfare and local public health centers. We also examined the relationship between railway-based population mobility and SIR.

By standardizing for age, we were able to better assess the impact of COVID-19 across different regions of Hokkaido, revealing notable disparities in SIR. Previous studies have shown that infection risk tends to be higher in areas with greater population density, and our findings are consistent with this trend [17-19]. Cities with higher density, such as Sapporo, Otaru, and Asahikawa, generally exhibited higher SIR values. The fluctuation in SIR values across pandemic waves may reflect differences in local public health interventions, behavioral responses, and sociodemographic characteristics. For instance, the elevated SIRs observed in Otaru and Asahikawa during the third wave, followed by a marked decrease in the fourth wave, may suggest the effectiveness of targeted interventions or increased community awareness after initial outbreaks. The resurgence of SIR in these cities during the fifth wave could be related to increased travel and social interactions during the summer season, coinciding with broader nationwide case surges. In the sixth wave, SIR became more uniform across urban regions, likely reflecting the widespread transmissibility of the Omicron variant, which may have overridden regional differences in exposure or control efforts. In contrast, the persistently low SIR observed in Hakodate and Other Areas suggests a relatively lower transmission risk, potentially due to lower population density, smaller household sizes, less interregional mobility, or earlier containment measures. These interpretations must be approached with caution, as the observed associations do not imply causation. 

Nonetheless, the updated regression models provided statistically significant evidence that railway transport density is associated with higher COVID-19 incidence. The significance of both railway transport density and Rt in the multivariable model suggests that intercity mobility and epidemic dynamics jointly shape regional disease burden. The negative coefficient for Rt may reflect the temporal mismatch between short-term transmission rates and cumulative incidence. These results highlight the utility of transport-related metrics as predictors in epidemic surveillance and planning. The negative association observed between Rt and SIR in the multivariable model should be interpreted with caution. While counterintuitive at first glance, this may reflect the temporal mismatch between Rt (a dynamic, real-time measure of transmission) and SIR (a cumulative incidence indicator). For example, areas with historically high transmission may have subsequently implemented stricter interventions or experienced behavioral changes, thereby reducing current Rt values. Further analysis using time-series or lagged regression models would be needed to clarify these dynamics.

Analysis of age-specific incidence rate ratios in these cities showed distinct differences in high-risk groups. In Sapporo, older adults consistently showed a higher risk of infection throughout the study period. One possible explanation is the relatively high number of elderly care facilities in Sapporo, compared to the national average, which may have contributed to sporadic cluster outbreaks. In contrast, Otaru showed peak incidence among individuals in their 20s to 50s during the third, fifth, and sixth waves. A similar pattern was observed in Asahikawa during the third and fifth waves. These trends may reflect the effects of commuting to and from larger urban centers such as Sapporo.

There are several limitations to this study. First, age-unreported cases were redistributed based on national age-specific incidence rates, which may not accurately reflect the actual age distribution. Additionally, we defined the susceptible population as the total population in each region, without adjusting for differences in vaccination coverage or changes in immunity over time. Factors such as inter-regional mobility, variations in local healthcare infrastructure, and other modes of transportation (e.g., air travel and road traffic) were not included in the analysis. Furthermore, since this study focused exclusively on Hokkaido, its generalizability to other regions of Japan may be limited. These limitations also highlight opportunities for future research. In particular, expanding the analysis to include additional transportation modes may enhance our understanding of mobility-related transmission dynamics. Future studies should incorporate additional modes of transportation, such as air travel and road traffic, to more comprehensively assess the impact of population mobility on infectious disease dynamics. While this study focused on railway transport, other travel routes may play a significant role in regions with limited rail connectivity or high rates of tourism and long-distance commuting. Integrating multi-modal transportation data with demographic and behavioral variables could yield a more complete picture of how mobility influences transmission patterns. Additionally, future analyses should consider dimensionality reduction techniques to address multicollinearity among demographic factors and explore models that incorporate spatial and temporal correlations across regions. These enhancements would improve the robustness of causal inference and predictive modeling in pandemic contexts. Furthermore, real-time mobility data and mobility network modeling may enhance the ability to predict outbreak hotspots and support the design of geographically tailored interventions in future pandemics.

## Conclusions

This study demonstrated that intercity railway mobility was positively associated with regional disparities in COVID-19 incidence in Hokkaido, Japan, even after adjusting for age structure. A key strength lies in the use of SIR and the integration of transport data, which provided a nuanced understanding of local transmission dynamics. Statistically significant associations between railway transport density and COVID-19 incidence were confirmed in both simplified univariable and multivariable regression models. These findings provide quantitative support for the hypothesis that intercity railway mobility contributes to elevated case burdens in highly connected urban areas. However, as this analysis is correlational, it does not establish a causal relationship. The results should be viewed as suggestive rather than prescriptive and may inform, but not determine, policy considerations.

The limitations of this study include the small sample size, the presence of multicollinearity among demographic variables, and the exclusion of other transportation modes such as road and air traffic. These findings underscore the importance of incorporating geographic and mobility-related factors into public health strategy and highlight the need for future studies using larger datasets and multimodal transport indicators to more comprehensively assess the impact of human movement on infectious disease transmission.
